# Insights into Cell Surface Expression, Supramolecular Organization, and Functions of Aquaporin 4 Isoforms in Astrocytes

**DOI:** 10.3390/cells9122622

**Published:** 2020-12-07

**Authors:** Jernej Jorgačevski, Robert Zorec, Maja Potokar

**Affiliations:** 1 Laboratory of Neuroendocrinology—Molecular Cell Physiology, Institute of Pathophysiology, Faculty of Medicine, University of Ljubljana, Zaloška 4, 1000 Ljubljana, Slovenia; jernej.jorgacevski@mf.uni-lj.si (J.J.); robert.zorec@mf.uni-lj.si (R.Z.); 2 Celica Biomedical, Tehnološki park 24, 1000 Ljubljana, Slovenia

**Keywords:** aquaporin 4 (AQP4), astrocyte, glia, orthogonal arrays of particles (OAP), brain edema, cell migration, extracellular matrix, hormones

## Abstract

Aquaporin 4 (AQP4) is the most abundant water channel in the central nervous system (CNS). Its expression is confined to non-neuronal glial cells, predominantly to astrocytes that represent a heterogeneous glial cell type in the CNS. The membrane of astrocyte processes, which align brain capillaries and pia, is particularly rich in AQP4. Several isoforms of AQP4 have been described; however, only some (AQP4a (M1), AQP4 c (M23), AQP4e, and AQP4ex) have been identified in the plasma membrane assemblies of astrocytes termed orthogonal arrays of particles (OAPs). Intracellular splicing isoforms (AQP4b, AQP4d, AQP4f, AQP4-Δ4) have been documented, and most of them are postulated to have a role in the cell surface distribution of the plasma membrane isoforms and in the formation of OAPs in murine and human astrocytes. Although OAPs have been proposed to play various roles in the functioning of astrocytes and CNS tissue as a whole, many of these still need to be described. OAPs are studied primarily from the perspective of understanding water permeability regulation through the plasma membrane and of their involvement in cell adhesion and in the dynamics of astrocytic processes. This review describes the cellular distribution of various AQP4 isoforms and their implications in OAP assembly, which is regulated by several intracellular and extracellular proteins.

## 1. Cellular Localization of Aquaporin 4 in Astrocytes

Aquaporin 4 (AQP4) is one of the three types of aquaporins (AQPs) that have been described in astrocytes [1]. Besides AQP4, astrocytes also express AQP1 and aquaglyceroporin AQP9, although in human astrocytes, the expression of the latter two is mainly detected in neuropathologic conditions [1]. Most AQPs transport only water molecules, as is the case with AQP1 and AQP4, but some, including AQP9, can also transport various non-charged solutes, including polyols, purines, pyrimidines, urea, and monocarboxylates [1,2,3,4]. AQP4 is the predominant water channel in the brain, and its expression is mainly confined to astrocytes, but its distribution and levels of expression vary between different brain regions [3]. In the cerebral cortex, AQP4 expression is particularly abundant in astrocytes aligning blood vessels [3]. A similar distribution along capillaries was detected throughout the hippocampus, corpus callosum, cerebellum, and brain stem where AQP4 labeling was confirmed around neuronal cell bodies [3,5]. Certain regions, such as osmosensory areas, do not show polarization of AQP4 expression [6]. Nevertheless, the predominant pattern of AQP4 in the central nervous system (CNS) is highly polarized, especially in astrocytes in the proximity of or in contact with blood vessels, the ependymal layer, and pia. Here, AQP4 is not distributed evenly in the plasma membrane; rather it is restricted to the plasma membrane of astrocyte endfeet that are in contact with the aforementioned structures [6,7]. Within these regions, AQP4 has a tendency to aggregate into higher order assemblies, termed orthogonal arrays of particles (OAPs).

### 1.1. Arrangement of AQP4 in Orthogonal Arrays of Particles

OAPs or AQP4 supramolecular assemblies in the plasma membrane of astrocytes can be readily detected experimentally by neuromyelitis optica (NMO) IgG antibodies applied to impermeabilized cells [8]. NMO IgGs are specific marker autoantibodies that are generated in patients with NMO and bind to the extracellular epitope of AQP4 [9,10,11]. Recently, it was revealed that a conformational epitope of AQP4ex (i.e., extended isoform of either AQP4a or AQP4c) appears to be critical for the binding of pathogenic human NMO-IgG autoantibodies to the perivascular astrocyte endfeet in the brain [12]. The commercially available AQP4 antibodies can label AQP4 only in permeabilized classically immunolabeled cells because they bind to intracellular epitopes [8]. In isolated murine astrocytes, both types of antibodies were tested, and only NMO-IgG labeling revealed typical plasmalemmal OAPs that were first identified by electron microscopy and at that time described as square arrays [8,13,14,15]. OAPs are morphologically diverse structures, composed of AQP4 tetramers that represent ~240 kDa building blocks that build higher order OAPs that can reach an approximate molecular weight of ~1000 kDa [16]. The reported OAP size expressed as the diameter varies between 100 and 500 nm. These values depend on several parameters, such as the microscopy technique of choice, whether the endogenous or recombinant OAPs were measured, and the abundance of a particular isoform within the OAPs. The diameters of endogenous OAPs in cultured neonatal rat astrocytes were between 150 and 650 nm (average ~300 nm) when imaged by total internal reflection fluorescence microscopy [14,15], ~170 nm when observed by structured illumination super-resolution microscopy [8], and their area covered 2142 ± 829 nm^2^ (which corresponds to a circle with a diameter of ~52 nm, or a square with a side length of ~46 nm) when recorded by direct stochastic optical reconstruction microscopy [17]. Recently, it was discovered that AQP4 undergoes translational read-through characterized by a C-terminal extension of translated AQP4 protein, which is an evolutionarily highly conserved trait [18]. In the cerebrum and cerebellum, AQP4ex displays perivascular polarization, and it was demonstrated in U87 cell lines that AQP4ex localizes to the plasma membrane where it aggregates in OAPs [19]. The size of OAPs containing AQP4ex isoforms AQP4-M1ex and AQP4-M23ex [19] have not been measured yet. OAPs formed only from AQP4-M23ex show a reduced tendency for clustering in OAPs, implying a functional role of the C terminus extension in OAP formation and dynamics [19].

### 1.2. Cellular Distribution of AQP4 in Astrocytes

**Figure 1:**
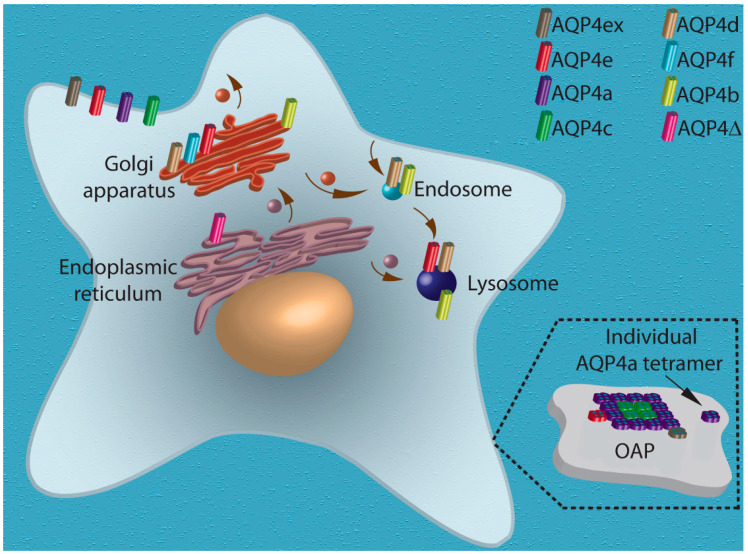
Cellular distribution of known AQP4 isoforms in astrocytes. Nine AQP4 isoforms have been identified in astrocytes: a (M1), b, c (M23), d, e, f in rat astrocytes and a (My1), c (M23), ex (note that both AQP4a and AQP4c are also expressed in the extended form) and Δ4 in human astrocytes. AQP4 isoforms a, c, and ex were detected at the plasma membrane, e was detected at the plasma membrane and in intracellular compartments, and other isoforms are solely intracellular, co-localizing with the Golgi apparatus (b, d, f), endoplasmic reticulum (Δ4), early endosomes (b, d), and lysosomes (b, d). Plasma membrane isoforms aggregate in OAPs. A patch of the plasma membrane containing OAPs and an individual AQP4a tetramer is enlarged in the dotted section. The core isoform in OAPs is isoform c (M23), isoform a (M1) assembles into OAPs through interlinking to c (M23). OAP localization of isoform e has been demonstrated in rat astrocytes and OAP localization of isoform ex has been demonstrated in human astrocytes. Isoform a (M1) is also present at the plasma membrane as individual tetramers, as depicted in the inset.

In addition to the predominant plasma membrane distribution, AQP4 isoforms are also distributed intracellularly, as shown by several studies on isolated murine astrocytes [8,13,20,21,22]. Whether at least some of them also have an intracellular distribution in the intact tissue, and to what extent, remains to be resolved. Immunochemical labeling in combination with forced expression of different recombinant AQP4 isoforms in isolated murine astrocytes revealed that they are distributed in the plasma membrane, as well as in distinct assemblies within the cytoplasm, resembling vesicular and organelle structures (Figure 1). For example, AQP4a (M1) and AQP4 c (M23), which were the first to be examined for their plasma membrane distribution and demonstrated to aggregate in clusters ~100 nm (AQP4c) and ~30 nm (AQP4a) in size [17], have never been investigated in detail for their possible cytoplasmic distribution. Of the nine AQP4 isoforms (AQPa–f, Δ4, AQP4[a and c]ex) [12,22], seven have been screened for their intracellular distribution in astrocytes. Among these seven isoforms, AQP4e and AQP4ex distribute to the plasma membrane, but AQP4e also shows a strong vesicular pattern in the cytoplasm [8,12,21]. Immunolabeling and vesicle mobility recordings in rat astrocytes revealed that AQP4e is expressed in highly dynamic vesicles, including endosomal compartments, and co-localizes also with the Golgi apparatus [21]. Similarly, in rat astrocytes and cell lines, AQP4b, AQP4d, and AQP4f isoforms co-localize with the Golgi apparatus and/or with early and late endosomal compartments; in addition, AQP4b and AQP4d have been detected in mobile vesicles [12,21,23]. AQP4-Δ4 isoform, first identified in skeletal muscle and brain, shows a broad cellular distribution with distinct localization in the endoplasmic reticulum of rat astrocytes, but not in the Golgi apparatus, with weak staining present in the plasmalemma [22]. In comparison with AQP4 isoforms located in the plasmalemma, knowledge of the role of exclusively intracellular isoforms is even more rudimentary. The roles of particular isoforms and their aggregations (OAPs) are reviewed in the following.

## 2. Regulation of OAP Size and Water Permeability by Distinct AQP4 Isoforms

### 2.1. Composition and Dynamics of OAPs

OAPs are aggregations of several AQP4 isoforms in the plasma membrane distinctively observed in astrocytes [24]. AQP4 monomers are first assembled into tetramers, which represent dynamic building blocks of OAPs that are highly variable in size, shape, and isoform composition [2,16,17,25]. For instance, one of the previously published models meticulously links the molecular interactions of different molar ratios of AQP4a and AQP4c with the OAPs [25]. These are not static structures, but mobile entities prone to constant rearrangements within the plasma membrane [26]. Isoform AQP4c (M23) has an inherent ability to spatially interact through the N terminus, and thus it forms the central core of OAPs; AQP4a (M1) can incorporate into OAPs only in the presence of isoform AQP4c [27,28,29], similar to AQP4e (Mz) [8,16,29]. A model based on experimental data suggest that the core of OAPs is formed by the heavily cross-bridged AQP4c (M23) isoform, and AQP4a (M1) attaches at the outer rim of OAPs, restricting its ability for further assembly and hence also confining its size and shape [25,27,28]. OAPs composed of only the AQP4c isoform are larger than those assembled from AQP4a and AQP4c combined [25,26,28,30,31,32]. On a similar note, the Mz isoform, expressed in rat but not in healthy human brain, impairs OAP size by affecting the ratio of AQP4c (M23)/AQP4a(M1) [33]. Adding to the complexity, OAPs are smaller in astrocytes devoid of AQP4ex [12]. Table 1 summarizes reported direct and indirect effects of respective AQP4 isoforms on OAPs.

Altered composition of the extracellular milieu can also affect OAP structure. Time-dependent changes in the size of OAPs were demonstrated in rat astrocytes overexpressing AQP4e exposed to hypoosmotic conditions mimicking brain edema [8]. In addition to the effect on OAP size, changes in the density of OAPs containing AQP4e were noted, signifying probable changes in insertion/retraction and redistribution of AQP4 isoforms within single OAPs [8]. Therefore, this particular isoform should be taken into account in models of OAP assembly in murine astrocytes. Movements of the other two plasma membrane isoforms have also been described. AQP4a (M1) appears to be more mobile within the plasma membrane, moving more freely, and AQP4c (M23) inclines to immobility within the plasmalemma [27,34].

### 2.2. Intracellular AQP4 Isoforms Can Affect the Organization of OAPs

In addition to plasma membrane movements, substantial rearrangements of AQP4 isoforms have been identified within the cytoplasm, with the potential to affect the arrangements of plasma membrane isoforms (see also Table 1). For instance, it was demonstrated that the abundance of AQP4 at the cell surface can be regulated by the AQP4-Δ4 isoform [22]. It was confirmed that the full-length AQP4 isoforms AQP4a and AQP4c interacted with truncated Δ4, which is retained in the endoplasmic reticulum, and therefore these complexes were targeted for degradation [22]. Regardless of this particular interaction, other interactions between AQP4 isoforms have not been confirmed in the endoplasmic reticulum. As demonstrated previously [26,35], OAPs do not assemble in the endoplasmic reticulum or in the Golgi apparatus. Obviously, for OAP assembly, we have to look at later stages in the secretory pathway and possibly also in the endosomal pathway, which at a certain point is intertwined with the secretory pathway. A recent study by Lisjak et al. [13] pointed out that the abundance of OAPs and their assembly is likely affected by early endosomal compartments containing different intracellular isoforms. The overexpression of AQP4b and AQP4d isoforms (splicing variants of AQP4a and AQP4c, respectively), which are abundantly located in the early endosomes but do not localize in the plasma membrane OAPs of rat astrocytes, reduced the abundance of OAPs. These changes coincided with altered intracellular trafficking of vesicles that transport particular isoforms and also with their substantial translocation into early endosomes in hypoosmotic conditions [8,13,21]. The role of early endosome-mediated regulation of OAP size in astrocytes is steadily emerging, and this organelle merits further attention also from this perspective.

### 2.3. OAPs and Water Permeability in Astrocytes

Water permeability through AQP4 channels in astrocytes may be regulated by several mechanisms and the role of OAPs in this is still not entirely clear. Altered expression levels of plasma membrane isoforms, delivery of isoforms via intracellular vesicles, phosphorylation of the channel, and rearrangement of isoforms within OAPs have been described among the processes that affect water permeability [1]. The role of AQP4 in the rapid regulation of astrocyte volume has been demonstrated for plasma membrane isoforms that localize to OAPs: AQP4c (M23) and AQP4e (Mz) [8,36]. An astrocyte cell line stably expressing AQP4c (M23) responded to hypoosmotic conditions with faster swelling kinetics, which in turn triggers regulatory volume decrease (RVD) of cells, a fast process that enables the original cell volume to be restored in response to swelling [36]. This kind of response is crucial for the regulation of cell volume in physiologic conditions where constant changes in intracellular and extracellular osmolarity induce cell swelling or shrinkage [37]. Similarly, overexpression of AQP4e in primary rat astrocytes also enhanced the kinetics of cell swelling and of RVD in comparison with non-transfected cells [8]. Although the plasma membrane isoforms were expected to affect water permeability across the plasmalemma, including the newly discovered AQP4ex isoform [19], this was not expected in the case of intracellular isoforms.

Therefore, one of the most conspicuous findings in recent years is the discovery that intracellular isoforms may affect the assembly and abundance of OAPs and consequently water homeostasis of astrocytes. When the truncated intracellular AQP4-Δ4 isoform, which is not a functional water channel, was co-transfected with the full-length AQP4, cells showed diminished water transport activity due to a reduction in the plasma membrane expression of AQP4, triggered by a dominant-negative effect as a consequence of heterodimerization between AQP4 and AQP4-Δ4 in the endoplasmic reticulum [22]. Furthermore, purely intracellular AQP4d has been shown to indirectly affect astrocyte volume changes, because astrocytes overexpressing AQP4d responded to the hypoosmotic milieu with faster swelling whereas RVD remained unaffected [13].

## 3. Extracellular Matrix and Intracellular Plasma Membrane-Associated Proteins Regulate Positioning of OAPs

**Figure 2:**
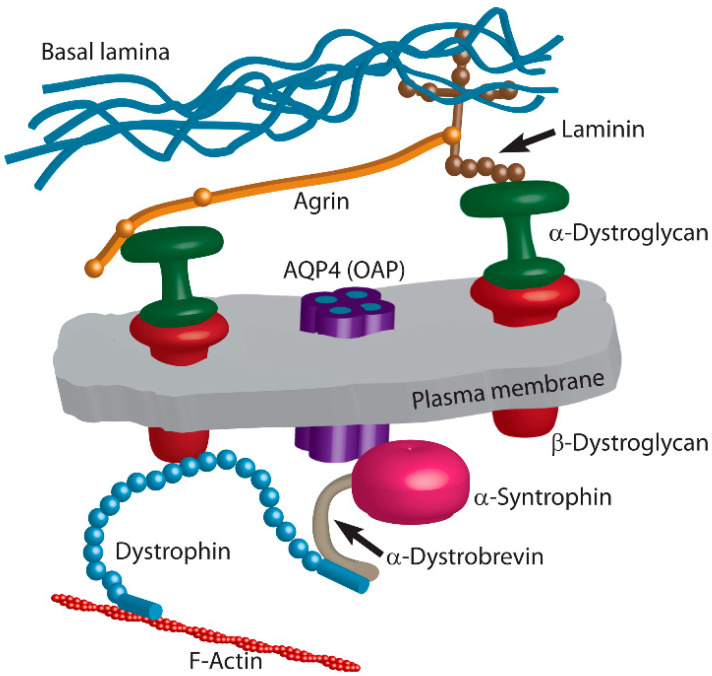
Positioning of individual AQP4 tetramers and OAPs in astrocytes is affected by intracellular and extracellular proteins. Agrin and laminin, components of the basal lamina, attach to α-dystroglycan, which forms a complex with β-dystroglycan that stretches through the plasma membrane. In the cytoplasm, it interacts with dystrophin, which binds to F-actin at one end and to α-dystrobrevin at the other end. α-Dystrobrevin interacts with α-syntrophin, which is directly linked to the AQP4 channel in the plasma membrane.

Positioning of individual AQP4 channels and OAPs in the plasma membrane of astrocytes has been receiving increasing attention in recent years, especially in connection with extracellular matrix proteins and proteins that directly or indirectly bridge the plasma membrane and AQP4 (Figure 2). While this is a highly relevant topic of research also in the view of numerous pathologies, it is still a developing field and recent data are described below.

### 3.1. Agrin

Agrin, an extracellular matrix protein, is a heparan sulfate proteoglycan and is a component of the basal lamina of blood-brain barrier (BBB) microvessels. It has been shown to be implicated in mechanisms that define the plasma membrane distribution of AQP4. This role of agrin was first described in human glioblastoma samples [38,39] and was further addressed in a mouse model, where differences in the appearance of OAPs were demonstrated between wild-type and agrin-devoid mice. Although astrocytes lacking agrin express OAPs in their endfeet, these are smaller, less dense, and, at certain places in astrocyte membranes, they were also absent [40,41]. This effect of agrin is not a consequence of altered overall expression of AQP4, because neither AQP4 mRNA nor AQP4 protein levels were changed in agrin-null astrocytes [41]. Moreover, in agrin-null astrocytes, the level of AQP4c (M23) protein expression remained higher than the expression of AQP4a (M1), which is similar to wild-type astrocytes [40,41]. The reduced abundance of OAPs in agrin-devoid astrocytes detected by immunocytochemical staining is therefore solely the consequence of post-translational processes.

The presence of exogenous agrin has been described to affect changes in cell volume in hypoosmotic conditions [41]. Astrocytes, wild-type or those devoid of agrin, responded to hypotonic stress with an increase in cell volume when they were grown on coating medium containing the neuronal agrin isoform A4B8 but not when grown on coating medium devoid of agrin [41]. Wild-type astrocytes expressing agrin showed a lower increase in cell swelling that agrin-null astrocytes, and fewer and smaller OAPs were detected [41]. These data are in agreement with the increasing evidence that the size and dynamic reorganization of OAPs play important roles in the water permeability of astrocytes, and further strengthen the hypothesis that AQP4 expression and OAPs are important for cell volume regulation in hypoosmotic conditions [8,36].

Although agrin acts as a key molecule important for increased density of OAPs in astrocyte endfeet [38], it is only one of the proteins involved in the polarity and clustering of OAPs in astrocytes (Figure 2).

### 3.2. Laminin

Laminin, another of the main structural proteins of the perivascular basal lamina, has also been demonstrated to induce plasma membrane clustering of not only AQP4 but also β-dystroglycan and Kir4.1 channels in hippocampal and cortical astrocytes [42,43]. Coating of growth substrate with laminin affects the abundance of AQP4 expression at the plasmalemma, without affecting the size of OAPs [21,42]. Increase in the abundance of AQP4 in the plasma membrane, in particular of the AQP4c (M23) isoform, is proposed to be regulated by dynamic redistribution from the plasma membrane, specifically through inhibited entry into early endosomal compartments [44]. Laminin apparently reduces the endocytosis of plasma membrane-localized principal OAPs forming AQP4c (M23) but not of AQP4a (M1) [44]. Laminin affects astrocyte plasmalemma in many ways; laminin was also shown to alter branching of astrocyte processes in cell cultures [45]. The branching process is proposed to be closely governed by direct and indirect interlinking of AQP4 on the laminin-dystroglycan-α-syntrophin axis [45]. Moreover, the suppressed expression of APQ4 per se also affected the formation of astrocyte processes, in particular the formation of second-order processes, via focal adhesion kinase phosphorylation [45].

In vivo studies supported the data obtained in cultured cells by showing that interaction between perivascular laminin and α-dystroglycan enables AQP4 positioning in perivascular astrocyte endfeet, which is also supported by a similar distribution of AQP4, α-syntrophin, and dystrophin–dystroglycan complex [46,47]. The effect of laminin on the positioning of agrin in hippocampal rat astrocytes, where laminin induced trafficking of endogenous agrin to the plasma membrane, also triggered clustering of AQP4 and β-dystroglycan [43]. Neither laminin nor agrin affect the overall expression of AQP4a (M1) or AQP4c (M23) isoforms, nor their ratio of expression [43], suggesting that clustering into OAPs and their reorganization depend on their recruitment from the cell cytoplasm to the cell surface and vice versa. The induced clustering of AQP4 triggered by addition of extracellular laminin into growth medium significantly attenuated astrocyte swelling in hypoosmotic conditions [43]. Again, this finding corroborates the hypothesis that OAPs regulate the propensity of astrocytes to the extent of cell volume changes. The size of OAPs in hypoosmotic conditions was not measured after laminin-induced AQP4 clustering [43], but the size of OAPs was determined in primary cultured rat cortical astrocytes, where they were more abundant and larger in hypoosmotic conditions [8,13]. The effects of laminin on AQP4 localization and function are in part relayed through protein kinase C signaling in lipid rafts [48].

### 3.3. Dystrophin–Dystroglycan Complex

#### 3.3.1. Dystroglycan

Agrin and laminin interact with a plethora of transmembrane proteins and cytoplasmic proteins that dynamically interact with the plasma membrane protein pool. Among these proteins is dystrophin–dystroglycan complex, composed of α- and β-dystroglycan, which is crucially involved in the plasma membrane localization of OAPs in astrocytes. Whereas α-dystroglycan binds to agrin and laminin [38,43,46,49], a plasma membrane-spanning β-dystroglycan directly and indirectly links dystroglycan complex to the cytoskeleton and other intracellular components, such as dystrophin and α-syntrophin [38,50]. These interactions further increase the number of components that may directly or indirectly contribute to OAP positioning in astrocytes.

One of the important steps in confirming the participation of α-dystroglycan in the localization of OAPs comes from experiments in which OAPs positively co-stained for α-dystroglycan and agrin [38,41]. Deletion of the dystroglycan gene reduces the expression of AQP4 protein in parenchymal brain tissue and causes a selective loss of OAPs in perivascular astrocytes together with disorganization of the membrane architecture of astrocyte endfeet [50]. In pathologic conditions, the expression of dystroglycan molecules may also decrease. For example, in human glioblastomas, the expression of α-dystroglycan is strongly reduced [51] and may coincide with decreased expression of agrin and increased expression of matrix metalloproteinases 3 and 2/9 [52]. Loss of the extracellular matrix receptor β-dystroglycan also leads to deficient AQP4 polarization in astrocytic endfeet [53]. Displaced localization of OAPs is proposed to be mediated by the loss of astrocyte endfeet anchoring to the basement membrane, which may even lead to edema formation, as demonstrated in experimental autoimmune encephalomyelitis, an animal model of multiple sclerosis [53]. However, in the absence of dystroglycans, a lower number of perivascular AQP4 molecules clustered into a few OAPs, implying dystroglycan-independent mechanisms in OAP positioning. Intuitively, one of the first assumptions would predict that the ratio of expression between the two most abundant plasma membrane isoforms, AQP4c (M23) and AQP4a (M1), was altered in dystroglycan-null mice. However, the protein ratio between the two was similar in wild-type and dystroglycan-null brain [50]. Therefore, one needs to take into account post-transcriptional changes in AQP4 isoforms that facilitate OAP formation and positioning, including other proteins that interlink with dystroglycan directly or indirectly. Apparently, further research is needed to resolve the various roles of dystroglycans in the formation and positioning of OAPs.

#### 3.3.2. Dystrophin

Dystrophin, a component of the multi-protein dystrophin–dystroglycan complex, is a cytoplasmic protein connected directly to β-dystroglycan via its amino terminal domain [54,55,56] (Figure 2). In humans, reduction in AQP4 expression was detected in muscle samples from patients with muscular dystrophy with different mutations in the dystrophin gene [57]. Similarly, dystrophin-deficient mice displayed reduced AQP4 expression in astrocytic endfeet surrounding capillaries [58,59]. The hypothesis has been proposed that dystrophin is important for stabilizing the whole multi-molecular complex that affects AQP4 positioning in the plasma membrane [38]. By affecting the positioning and formation of OAPs [38], the function of dystrophin appears to be associated with swelling of astrocytic perivascular processes and consequently with brain edema [58]. The shorter isoform of dystrophin, glial dystrophin isoform Dp71, is the major dystrophin gene product responsible for anchoring AQP4 and the dystroglycan complex at the glial endfeet [15,60,61]. Even though interactions of AQP4 with a dystrophin-dependent pool have been predominantly investigated thus far, dystrophin-independent pools detected in the endfeet of subpial astrocytes and in granular layer astrocytes in the cerebellar cortex imply that additional proteins may be involved in OAP positioning in astroglia membranes [60].

#### 3.3.3. α-Syntrophin

Dystrophin interlinks (likely indirectly) with intracellular protein α-syntrophin [62], and this connection contributes to complex interactions that regulate the positioning of OAPs in astrocytes. The involvement of α-syntrophin in the distribution of AQP4 in astrocytes was first postulated from an observation in α-syntrophin-null mice whereby perivascular and subpial astrocyte endfeet in the cerebral cortex and cerebellum showed markedly reduced labeling of AQP4 [63]. Further corroboration of the importance of α-syntrophin in the positioning of OAPs in the plasma membrane of astrocytes came by demonstrating direct interaction of the AQP4 C terminus to α-syntrophin [59,63,64,65]. Prominent perivascular endfeet co-localization of AQP4 and α-syntrophin was also demonstrated in rat brain [38]. Furthermore, the association between α-syntrophin and AQP4 was detected in AQP4 transfected U87MG human glioblastoma cells, which natively do not express AQP4 [7], and in human astrocyte cultures [35].

In addition to AQP4c (M23), which was primarily proposed to be the linking partner of α-syntrophin [65], AQP4ex was described recently and found co-localized with α-syntrophin in HEK cells and mice [19,23]. In a CRISPR-Cas9-generated AQP4ex^−/−^ mouse model, the prominent localization of AQP4 in astrocyte endfeet was compromised simultaneously with decreasing expression and cortical mislocalization of α-syntrophin [12]. Although the absence of α-syntrophin in α-*Syn*^−^^/^^−^ mice does not affect the total levels of AQP4c (M23) and AQP4a (M1) expression in the brain, the polarization pattern of AQP4 in the astrocytic endfeet adjacent to the endothelial basal lamina in the cerebral cortex is significantly diminished in astrocytes devoid of α-syntrophin [63,64,66]. On the same note, prominent AQP4 polarization in perivascular endfeet vanishes in various pathologic conditions, such as human glioblastoma, where AQP4 redistributes throughout the cell membrane together with α-syntrophin [38].

The lack of α-syntrophin expression affects water regulation properties of astrocytes, which further substantiates the proposition that abundant localization of AQP4 in perivascular endfeet of astrocytes is essential for the regulation of water homeostasis in the brain. Namely, perivascular and subpial astrocyte endfeet have a swollen appearance in the brain of α-syntrophin-null mice, suggesting that clearance of water in the brain is reduced [64]. Further studies on α-syntrophin-null mice revealed that the AQP4 pool in perivascular astrocytes may control the rate of osmotically driven water entry [66]. In α-syntrophin-null mice, in which expression of the perivascular pool of AQP4 was specifically abolished, a delay in the development of hypoosmotic brain edema was recorded [66].

## 4. OAPs and Astrocyte Migration

### 4.1. OAPs in Migration and Adhesion of Astrocytes

The involvement of AQP4 expression in the potential for astrocyte migration was first observed in cultured cortical astrocytes, where astrocytes devoid of AQP4 showed greatly impaired migration compared with their wild-type counterparts [67]. At the same time, this finding was corroborated by in vivo stab injury experiments where reactive astrocytes in AQP4-null mice migrated more slowly toward the stab lesion [67,68]. Moreover, accelerated migration potential of astrocytes toward hypo-osmolar medium was proposed to be related to AQP4-linked increased plasma membrane permeability, which upsurges the transmembrane water fluxes that take place during cell movement [67].

Processes that affect the migration potential of astrocytes are complex and related to interactions of AQP4 tetramers with the cytoskeleton, as demonstrated in astrocytoma cells [69]. An important notion about the data obtained in astrocytoma cells is that, when they are grown in culture for several passages, they greatly decrease the expression of AQP4; but when AQP4 is reintroduced, these cells exhibit enhanced cell adhesion potential [70], which is an important feature in cell migration. Furthermore, the migration potential of astrocytes appears to be affected differently by the aggregation potential of particular AQP4 isoforms. Namely, the aggregation state of particular AQP4 isoforms had an important effect on their subcellular localization in cultured *AQP4^−/−^* cortical astrocytes and glioblastoma cells [71]. For instance, AQP4a (M1), which is not able to assemble into OAPs per se and has the ability to move freely in the plasma membrane, was detected not only in the soma of astrocytes but also extensively in lamellipodia [71]. Enriched presence of AQP4a (M1) retained a more stable and longer-lasting shape of lamellipodia, which facilitated astrocyte migration [71]. The leading edge of migrating cells with abundant AQP4a (M1) is also the site of transmembrane osmotic pressure gradients generated by activation of ion channels or exchangers [71]. On the other hand, AQP4c (M23), which forms large OAPs, is localized in the cell body and endfeet of pericapillary astrocytes, seemingly does not affect lamellipodia stability, but appears to be important for cell adhesion and polarization processes [71]. Together with isoform-specific changes in AQP4 localization and redistribution of AQP4 in high-grade astrocytomas, mislocalization of inwardly rectifying K^+^ channel Kir4.1 was detected in low- and high-grade astrocytomas and oligodendrogliomas [72]. This finding suggests that, in these cells, the buffering capacity of glial cells may also be compromised, leading to water influx and causing cytotoxic edema [72]. If the mechanisms of redistribution of AQP4 and Kir4.1 differ in low- and high-grade gliomas, this may suggest that the mechanisms of clustering of AQP4 and Kir4.1 at the glial endfeet membrane domains are also different [72].

### 4.2. OAPs and Glioma Invasiveness

Distinct localization patterns of AQP4 isoforms that are present in the human brain [71] imply that different AQP4 isoforms may serve diverse purposes, including astrocyte mobility. Recent research has shown that localization and the aggregation state of AQP4a (M1) and AQP4c (M23) most likely participate in the fate of glioma cells, where the two isoforms probably play different roles to either enhance glioblastoma invasiveness or cease their proliferation potential [69]. Selective expression of AQP4a (M1) enhanced migration of glioma cell lines and increased matrix methalloproteinase-9 activity, the overexpression of which is enhanced in multiple neurodevelopmental disorders [69,73]. The expression of AQP4c (M23) directed glioma cells to apoptosis through interactions mediated by actin cytoskeleton, therefore reducing the invasive potential [69]. The protein expression of AQP4 in human glioblastoma is indeed upregulated in comparison with healthy brain tissue [52,74,75]. However, the ratio of expression between AQP4a (M1) and AQP4c (M23) appears to remain similar [52]; therefore, one has to look at additional levels that affect the incorporation of AQP4 into the plasma membrane and their interactions within OAPs. These are post-translational processes demonstrated to have the potential to modify certain stages of OAP formation, including delivery and retraction of isoforms to/from the plasma membrane, rearrangements within the plasma membrane, and yet poorly identified interactions with specific cellular organelles, such as the endoplasmic reticulum and early endosomes. Interestingly, the abundance and the size of OAPs differ not only in comparison with healthy cells but also between different stages of glioblastomas. High-grade astrocytoma tumors are reported to express only a few typical OAPs, whereas low-grade astrocytomas express more OAPs; this discovery is among the important steps in unraveling of the role of OAPs in astrocytoma malignancy [52,74]. Functional rearrangements of OAPs in glioblastomas remain to be further evaluated from the perspective of rearrangements of different AQP4 isoforms, because not all AQP4 molecules in the plasma membranes of glioblastoma cells are arranged in OAPs [52,71].

## 5. Neurotransmitters and Hormones That Modify AQP4 Expression

In addition to extracellular matrix proteins and proteins interlinking with the dystrophin–dystroglycan complex that guide positioning of OAPs in the plasma membrane of astrocytes, external stimuli may also affect OAP arrangements. In addition to changes in osmolality and other multifaceted processes that modify AQP4 expression and hence the distribution and the formation of OAPs in traumatized brain, hormones are the most prominent factors for alterations in AQP4 expression (Table 2) and possibly also for rearrangements of OAPs in astrocytes. Changes in AQP4 expression, as a consequence of hormonal activity, which can trigger a variety of physiologic responses in astrocytes, are described in the following sections.

### 5.1. Dopamine

Increased levels of dopamine have been demonstrated to reduce proliferation of striatal astrocytes and in downregulation of AQP4 expression [76]. However, these data need to be viewed with great caution, because cells growing more sparsely showed larger effects than densely plated cells. How this effect relates further to AQP4 functions in astrocytes in situ and in vivo remains to be investigated. One of the possible consequences of AQP4 downregulation is reduced water permeability through the plasma membrane, as observed in cultured rat cortical astrocytes [77], and reduced proliferation, as seen in cultured striatal mouse astrocytes [76]. Dopamine treatment of mouse striatal astrocytes selectively downregulated only the AQP4c (M23) isoform [76], which implies that altered dopamine levels may affect the abundance of OAPs in astrocytes. This, as well as the general relationship between dopamine and AQP4 expression and AQP4 function (i.e., water permeability, migration), remains to be further investigated. In addition, the role of AQP4 in the brain also needs to be addressed from an immunological perspective. The lack of AQP4 expression in the mouse model of Parkinson disease (PD) was shown to abolish an increase in transforming growth factor-β1 (TGF-β1) [78]. TGF-β1 is a key suppressive cytokine, playing a role in PD onset and development, and abolishing its increase in the midbrain triggers stronger microglial inflammatory responses and greater losses of dopaminergic neurons [78]. This effect was partially attributed to impaired generation of TGF-β1 by astrocytes lacking AQP4 expression [78]. The lack of AQP4 expression was also related to stronger astrogliosis in the midbrain of a PD mouse model triggered by administration of 1-metil-4-fenil-1,2,3,6-tetrahidropiridin (MPTP). At the same time, more prominent demise of the dopaminergic (DA) neurons was observed, showing that mice lacking AQP4 were more prone to MPTP-induced neurotoxicity than their wild-type littermates [79,80]. Moreover, it appears that the effect of AQP4 extends even further, acting more selectively. In human PD brain and in wild-type mice brain, differences in the degeneration of DA neurons are noticeable between the substantia nigra and the ventral tegmental area. These differences in degeneration of DA neurons between different brain regions disappear in AQP4 knockout mice [81].

### 5.2. Testosterone

Over the last decades, sex hormones have been increasingly recognized as important agents that affect the functioning of several cell types and their functions in the CNS. In certain cases of acute neurological injuries, the gender issue is important, as was observed in experimental models and in humans [89]. Of several sex hormones that have been studied in relation to AQP4 expression, testosterone is the least explored and has been demonstrated to dose-dependently increase the expression of AQP4 mRNA and protein in cultured rat cortical astrocytes [82]. Testosterone-related increase in AQP4 expression beneficially affected the osmotic fragility of astrocytes exposed to hypoosmotic stress [82], demonstrating potentially beneficial effects of increased testosterone related to the AQP4 channel. This effect could be related to alleviating cytotoxic edema by enhancing the RVD potential of astrocytes [82]. When testosterone-related changes in AQP4 expression were tested in testosterone-treated rats, an increase in AQP4 expression was measured at the basolateral membrane of kidney collecting ducts [90]. The level of AQP4 expression related to increased testosterone levels was not tested in the intact brain. Considering the important possible implications of testosterone action in the CNS, this topic needs further attention to establish the effect of testosterone on AQP4-related functions in astrocytes.

### 5.3. Progesterone

Progesterone is another sex hormone that can play an important role in several aspects of CNS insult, such as traumatic brain injury (TBI) and stroke, having the potential for treatment of TBI and other neural disorders in humans [91]. Progesterone has been demonstrated to have beneficial effects on the survival of neurons after focal cerebral ischemia [92,93]. These protective effects are related to a decrease in the pro-inflammatory responses [94] in which astrocytes crucially participate as regulators of innate and adaptive immune responses in the injured CNS [95]. Astrogliosis that is triggered after CNS injury was found to be decreased in the peri-infarct area of progesterone-treated mice, together with a decrease in AQP4 expression [83]. In rat astrocytes, progesterone attenuated AQP4 mRNA levels and protein expression and acted positively on cell viability in an astrocyte model of ischemia/reperfusion [84]. Positive effects of progesterone on brain damage were also demonstrated in neonatal rats with induced hypoxic ischemia; downregulation of AQP4 expression and MMP-9 in progesterone-treated animals had beneficial effect on alleviation of cerebral edema [96]. However, progesterone-induced changes in AQP4 expression must be interpreted with caution, taking into account the time after progesterone treatment and different brain regions, which may respond differently in the matter of AQP4 expression. In a model of TBI, it was demonstrated that progesterone administration decreased AQP4 expression around the contusion and in the tissue bordering the lateral ventricular area, whereas it increased AQP4 expression in the hypothalamic region with osmosensitive neurons [85]. These changes in AQP4 expression are possibly implicated in the formation and resolution of TBI-induced cerebral edema [85] and deserve further attention at the level of expression of different AQP4 isoforms.

### 5.4. Estradiol

Neuroprotective effects of estradiol have been reported in several studies. Protective actions of estradiol in neurodegenerative diseases and injuries are exerted by several complex mechanisms that regulate the proliferation and survival of cells and their anti-inflammatory responses [97]. Protective effects of estradiol may also be related to the abundance of AQP4 in astrocytes; however, further attention is needed in terms of stimuli, spatial and temporal mode of action of estradiol on AQP4 expression in astrocytes. On the one hand, increase in AQP4 expression in astrocytes was shown to be alleviated by long-term exposure to a higher dose of estradiol (1 week), whereas exposure to estradiol for several hours did not show any significant trend in AQP4 expression [86]. On the other hand, in a model of induced BBB disruption by intraparenchymal injection of lipopolysaccharide, the increase in expression of AQP4 in perivascular astrocytes was detected within several hours and was also lessened by treatment with estradiol [87].

Regarding altered AQP4 expression in the development and reabsorption of brain edema, several hypotheses have been proposed. On the one hand, the upregulation of AQP4 channels has been proposed as beneficial in relieving the early phase of vasogenic edema [98] and on the other hand, inhibition of AQP4 upregulation may dramatically improve clinical outcome in certain pathologic stages [99]. Clearly, changes in the expression of AQP4 affected by estradiol and other sex hormones should be considered by taking into account the appropriate time window and affected region in neurotrauma. For example, the effect of estradiol on the reduction of astrocyte swelling in hypoxia- and arginine vasopressin (AVP)-induced stress took place on an hourly basis, whereas changes in AQP4 expression followed in a matter of several days [86]. This is why the short-term effects of estradiol on rearrangement of AQP4 isoforms in the plasma membrane should also be considered and investigated. Current results suggest that the levels of AQP4a (M1) and AQP4c (M23) expression are reduced [86], but the ratio between the two isoforms in conditions with or without estradiol was not compared, which prevents us from stating a definite conclusion on the effects of estradiol on the abundance of AQP4 in the plasma membrane.

In addition to cell volume regulation and the development of brain edema, other roles of estradiol and other sex hormones of astrocyte function that are involved in recovery after neurotrauma (such as migration, adhesion, proliferation) should be considered.

### 5.5. Arginine Vasopressin

The effect of AVP on swelling of astroglia cells was described three decades ago when it was documented that AVP causes gradual persistent astroglial swelling [100]. Later, it was discovered that this effect of AVP-induced astroglia swelling can be blocked by V1 antagonist [101]. The discoveries that followed advanced our understanding of the function of the intrinsic vasopressin fiber system. Several studies demonstrated that AVP, acting via V1a receptors, plays a crucial role in the regulation of brain water and ion homeostasis [102], and V1a receptor is involved in the pathophysiology of secondary brain damage after focal cerebral ischemia [103]. Furthermore, V1aR inhibitors are considered potential therapeutic tools for treating cellular brain edema [104]. Inhibition of V1a receptors has been shown to considerably reduce AQP4 upregulation in contusion areas of traumatized brain [105]. This change was mirrored in reduced brain water content in the traumatized hemisphere in the presence of V1aR inhibitors [105]. Selective V1aR inhibition by the non-peptide antagonist decreased brain AQP4 expression, brain edema, and astrocytic cell swelling [106]. In induced hyponatremia (hypotonic dextrose and AVP), model rats showed increased immunoreactivity of AQP4, as observed in immunoblots in the cerebellum, whereas mRNA expression remained unaltered after several hours of exposure [88]. This observation may reflect secondary conformational modifications of AQP4 protein, leading to enhanced antibody binding and other post-translational modifications of AQP4 [88]. Although electron immunomicrographs failed to reveal any substantial changes in cellular localization of AQP4 [102], the possibility that vasopressin causes reorganization of the plasma membrane AQP4 molecules within the individual membrane compartments cannot be ruled out. Rearrangements of specific plasma membrane isoforms within and between OAPs should be considered in further studies.

Large extracellular ionic fluxes of Na^+^ and K^+^ that occur rapidly after focal brain injury may result in cellular edema [102]. Facilitation of water transport through astrocytic AQP4 in the presence of AVP allows cells to better compensate for the build-up of extracellular osmotic gradients by K^+^ release from neurons [102]. Water fluxes are extremely rapid, indicating that they are mediated by AQPs [102]. Thus, astrocytes fit perfectly with the hypothesis, supported by discovery in rat neocortical brain slices that the glial network plays an important role as a draining system for spatial buffering of extracellular K^+^ [107,108].

## 6. Conclusions

The increasing number of newly discovered AQP4 isoforms in astrocytes, combined with the additional roles proposed for those that have already been studied for an extended period of time, are advancing our knowledge about the various roles of AQP4 protein in the brain. Newly emerging data on AQP4 isoforms that have been identified to date are calling for reconsideration of some of the existing models of AQP4 functions. For example, in recent years, three additional plasma membrane isoforms have been discovered. One of them, AQP4e, was identified in rats and shown to localize in OAPs and to affect RVD in astrocytes. Therefore, an updated model of OAP structure in rat astrocytes, involving (at least) three plasma membrane isoforms (AQP4c [M23], AQP4a [M1], and AQP4e) needs to be designed. Two AQP4ex plasma membrane isoforms that were recently identified in human astrocytes and revealed as crucial for binding NMO IgGs were also shown to build OAPs. Hence, updated modeling of OAP organization and dynamics is also required for human astrocytes. In addition to plasma membrane AQP4 isoforms, recently described intracellular isoforms AQP4b, AQP4d, and Δ4, are also being revealed as modulators of the abundance and size of OAPs. New discoveries about particular isoforms in OAP assembly and abundance, the role of isoforms that are detected as single tetramers in the plasma membrane of astrocytes, and the role and dynamics of intracellular isoforms extend our view of AQP4 channels as regulators of water homeostasis of the brain. Cell adhesion and migration processes intimately linked to AQP4 isoforms are yet to be unraveled in normal physiologic and pathologic conditions, such as acute neurotrauma and neurologic diseases where particular isoforms and assembly in OAPs in the plasma membrane of astrocytes might affect the development and the outcome of the particular pathologic conditions. Several intracellular and extracellular matrix proteins are emerging as important factors for positioning and polarization of AQP4 tetramers and OAPs at the plasma membrane of astrocytes. The role of these proteins and their interactions with APQ4 channels in normal and pathologic conditions are still emerging and require further attention to understand the larger picture of the changes that are taking place in traumatized brain. Additionally, the consequences of hormone imbalance and the composition of the extracellular milieu affecting ion homeostasis are crucial and need to be investigated in further studies.

## Figures and Tables

**Table 1:** AQP4 isoforms affect the abundance and size of OAPs directly and indirectly.

AQP4 Isoform	Species/mRNA	Incorporation into OAPs	Reference
AQP4a (M1)	Human, rat, mouse/basic mRNA; posttranscriptional regulation defines the amount of protein in the plasma membrane	Yes (through interaction with AQP4c (M23))	[17,25,27,28,29,30,31,32]
AQP4a (M1) ex	Human, rat/extended mRNA through translational readthrough	Yes	[19]
AQP4c (M23)	Human, rat/basic mRNA; posttranscriptional regulation defines the amount of protein in the plasma membrane	Yes (it is the core OAP constituent)	[17,25,27,28,29,30,31,32]
AQP4c (M23) ex	Human, rat/extended mRNA through translational readthrough	Yes (modulates the size of OAPs by limiting the number of added tetramers)	[19]
AQP4e (Mz)	Rat/basic mRNA	Yes (through interaction with AQP4c (M23))	[8,23,29]
AQP4b	Rat/mRNA isoform lacking exon 2 after alternative splicing from AQP4a	No (indirect modulation of the abundance of OAPs)	[13,23]
AQP4d	Rat/mRNA isoform lacking exon 2 after alternative splicing from AQP4c	No (indirect modulation of the abundance of OAPs)	[13,23]
AQP4f	Rat/mRNA isoform lacking exon 2 after alternative splicing from AQP4c	Not tested	[23]
AQP4-Δ4	Human/mRNA isoform lacking exon 4 after alternative splicing from AQP4a	No (it may modulate the abundance and size of OAPs by dominant-negative effect exerted in endoplasmic reticulum through protein–protein interactions with the plasma membrane AQP4 isoforms)	[22]

**Table 2:** Neurotransmitters and hormones affecting AQP4 expression.

Hormone	AQP4 Expression	Astrocytes/Species	References
Dopamine	Downregulated	Striatal and cortical astrocytes/mouse	[76,78]
Testosterone	Upregulated	Cultured cortical astrocytes/rat	[82]
Progesterone	Downregulated or Upregulated (brain region specific)	Astrocytes in vivo, primary cultured astrocytes/mouse, rat	[83,84,85]
Estradiol	Downregulated	Cultured cortical astrocytes, brain slices/rat	[86,87]
Arginine vasopressin	Increased immunoreactivity, no changes in mRNA	Whole brain, cerebellum/Rat	[88]

## Abbreviations

AQP	aquaporin
AVP	arginine vasopressin
BBB	blood-brain barrier
CNS	central nervous system
MPTP	1-metil-4-fenil-1,2,3,6-tetrahidropiridin
NMO	neuromyelitis optica
OAP	orthogonal array of particles
PD	Parkinson disease
RVD	regulatory volume decrease
TBI	traumatic brain injury
TGF-β1	transforming growth factor-β1
